# Non-pharmacological treatment changes brain activity in patients with dementia

**DOI:** 10.1038/s41598-020-63881-0

**Published:** 2020-04-21

**Authors:** Yoshihito Shigihara, Hideyuki Hoshi, Keita Shinada, Toyoji Okada, Hajime Kamada

**Affiliations:** 1 Precision Medicine Centre, Hokuto Hospital, Obihiro City, Japan; 2 Department of Neurosurgery, Hokuto Hospital, Obihiro City, Japan; 3 Geriatric Health Services Facility Kakehashi, Hokuto Hospital Group, Obihiro City, Japan; 4 Department of Clinical Laboratory, Hokuto Hospital, Obihiro City, Japan

**Keywords:** Personality, Rehabilitation

## Abstract

Non-pharmacological treatment (NPT) improves cognitive functions and behavioural disturbances in patients with dementia, but the underlying neural mechanisms are unclear. In this observational study, 21 patients with dementia received NPTs for several months. Patients were scanned using magnetoencephalography twice during the NPT period to evaluate NPT effects on resting-state brain activity. Additionally, cognitive functions and behavioural disturbances were measured using the Mini-Mental State Examination (MMSE-J) and a short version of the Dementia Behaviour Disturbance Scale (DBD-13) at the beginning and the end of the NPT period. In contrast to the average DBD-13 score, the average MMSE-J score improved after the NPT period. Magnetoencephalography data revealed a reduced alpha activity in the right temporal lobe and fusiform gyrus, as well as an increased low-gamma activity in the right angular gyrus. DBD-13 score changes were correlated with beta activity in the sensorimotor area. These findings corroborate previous studies confirming NPT effects on brain activity in healthy participants and people at risk of dementia. Our results provide additional evidence that brains of patients with dementia have the capacity for plasticity, which may be responsible for the observed NPT effects. In dementia, NPT might lead to improvements in the quality of life.

## Introduction

Dementia is a chronic and progressive syndrome caused by brain diseases^1^. It is characterised by deterioration in cognitive functions, behaviour, and psychological symptoms. To date, there are only a few pharmacological treatment options available to influence the course of dementia^2–4^. At the time a patient is diagnosed with dementia, the brain damage is considered too severe to be reversible to a healthy state^5^. Thus, early diagnosis and interventions are essential to treat people at risk of dementia, such as those with “cognitive impairment, no dementia” (CIND) or “mild cognitive impairment” (MCI)^6–9^. Recent studies showed that MCI can be reversed to normal cognitive functions by non-pharmacological treatments (NPTs)^10–15^. Neuroimaging studies revealed that NPTs enhance brain plasticity even in older adults^16,17^ supporting the importance of early diagnosis and interventions in dementia.

The positive effects of NPTs are not limited to people at risk such as those with CIND or MCI. Clinicians and therapists frequently observe that NPTs improve cognitive functions and/or behaviours in patients with dementia^18–23^. However, the neural mechanisms underlying the positive influences of NPTs remain unclear.

Geriatric health service facilities are transitional facilities between hospital and home or nursing home where registered physical, occupational, and speech therapists provide NPT and nursing care to reduce the patients’ hospital stay. Patients with dementia are often transferred there when their physical conditions are usually not too severe to be treated in hospitals but their cognitive symptoms prevent them from staying at their home. The primary goal of the facility is to improve the patients’ behavioural functions, to enable them to live again at home^21^. Although cognitive improvements are the secondary target, patients in these facilities often show progress in cognitive functions, as well as in behavioural and psychological symptoms of dementia (BPSD)^20^. Based on these experiences, clinicians and therapists are often under the impression that patients with dementia still exhibit sufficient brain plasticity to respond to NPTs; however, evidence for this is sparse in literature. In this study, 21 patients with dementia were recruited at our geriatric health service facility. They received NPTs for several months and their resting-state brain activities were measured using magnetoencephalography (MEG) two times during the total NPT period. The brain activity recordings were compared to better understand how NPT changes the resting-state brain activities in patients with dementia.

## Results

### Behavioural assessments

The average MMSE-J score was 12.4 ± 6.6 at the beginning and 14.4 ± 7.8 at the end of the NPT period (Table 1). The score increased (i.e. the cognitive level improved) in 10 patients, decreased in 5 patients, and remained unchanged in 3 patients. The mean score was significantly increased at the group level (*p* = 0.002, using bootstrap method). The changes in the MMSE-J score did not correlate with the patients’ age, duration of the NPT period, or the initial MMSE-J score. Taken together, the cognitive functions of the study population improved over the NPT period but the patients’ age nor their initial cognitive function was a predictor of this change.

**Table 1:** Patients’ profiles.

ID	Age	Sex	Reported	Interval of the scans	Initial score			Final score			Change		
			Diagnose	(days)	Severity	MMSE	DBD-13	Severity	MMSE	DBD-13	Severity	MMSE	DBD-13
Average	85.1			75.7	4.2	12.4	10.6	3.9	14.4	9.9	−0.2	2.0	−0.7
SD	5.4			31.7	1.6	6.6	10.8	1.6	7.8	11.1	0.5	3.2	2.7
Max	94.0			133	6.0	22.0	43.0	6.0	29.0	40.0	0.0	8.0	7.0
Min	73.0			28	2.0	0.0	0.0	2.0	0.0	0.0	−2.0	−3.0	−4.0
Median	84.5			75	4.0	12.5	6.5	4.0	12.5	5.5	0.0	2.0	−1.0
1	73	F	VD	73	3	18	0	2	20	0	−1	2	0
2	79	F	VD	77	6	3	4	6	5	3	0	2	−1
3	79	M	VD	52	4	8	0	4	13	2	0	5	2
4	80	F	VD	95	2	21	2	2	21	1	0	0	−1
5	81	F	Not specified	91	6	0	43	6	0	40	0	0	−3
6	83	F	AD	53	4	13	6	4	12	6	0	−1	0
7	84	M	AD	133	6	12	4	5	11	3	−1	−1	−1
8	84	F	AD	133	5	11	20	3	18	19	−2	7	−1
9	84	M	AD	95	2	21	4	2	24	1	0	3	−3
10	85	M	AD	31	5	7	10	5	6	11	0	−1	1
11	85	M	AD	98	6	11	23	6	11	30	0	0	7
12	89	F	AD + PD	28	3	14	5	3	17	4	0	3	−1
13	89	F	AD	98	3	14	14	3	11	17	0	−3	3
14	90	F	AD	70	4	14	3	4	16	1	0	2	−2
15	90	M	Not specified	70	6	9	21	6	8	17	0	−1	−4
16	91	M	AD	90	2	21	7	2	29	6	0	8	−1
17	91	M	AD	39	2	22	8	2	27	5	0	5	−3
18	94	F	AD	36	6	4	16	6	10	12	0	6	−4

VD, Vascular dementia; AD, Dementia due to Alzheimer’s disease; PD, Parkinson’s diseases.

The average DBD-13 score changed from 10.6 ± 10.8 at the beginning of the NPT period to 9.9 ± 11.1 during its end. This score decreased (i.e. behavioural problems were alleviated) in 12 patients, increased in 4 patients, and remained unchanged in 2 patients. The improvement of the DBD-13 score was not statistically significant (*p* = 0.132, using bootstrap method) at the group level. The change in the DBD-13 score did not correlate with age, duration of the NPT, or initial DBD-13 score.

The MMSE-J and DBD-13 scores were negatively correlated at the beginning (*p* = 0.010, using bootstrap method) and the end (*p* = 0.004, using bootstrap method) of the NPT period. However, the changes in MMSE-J and DBD-13 scores showed no statistically significant correlation (*p* = 0.084, using bootstrap method).

### Changes in spontaneous neural oscillations

**Figure 1:**
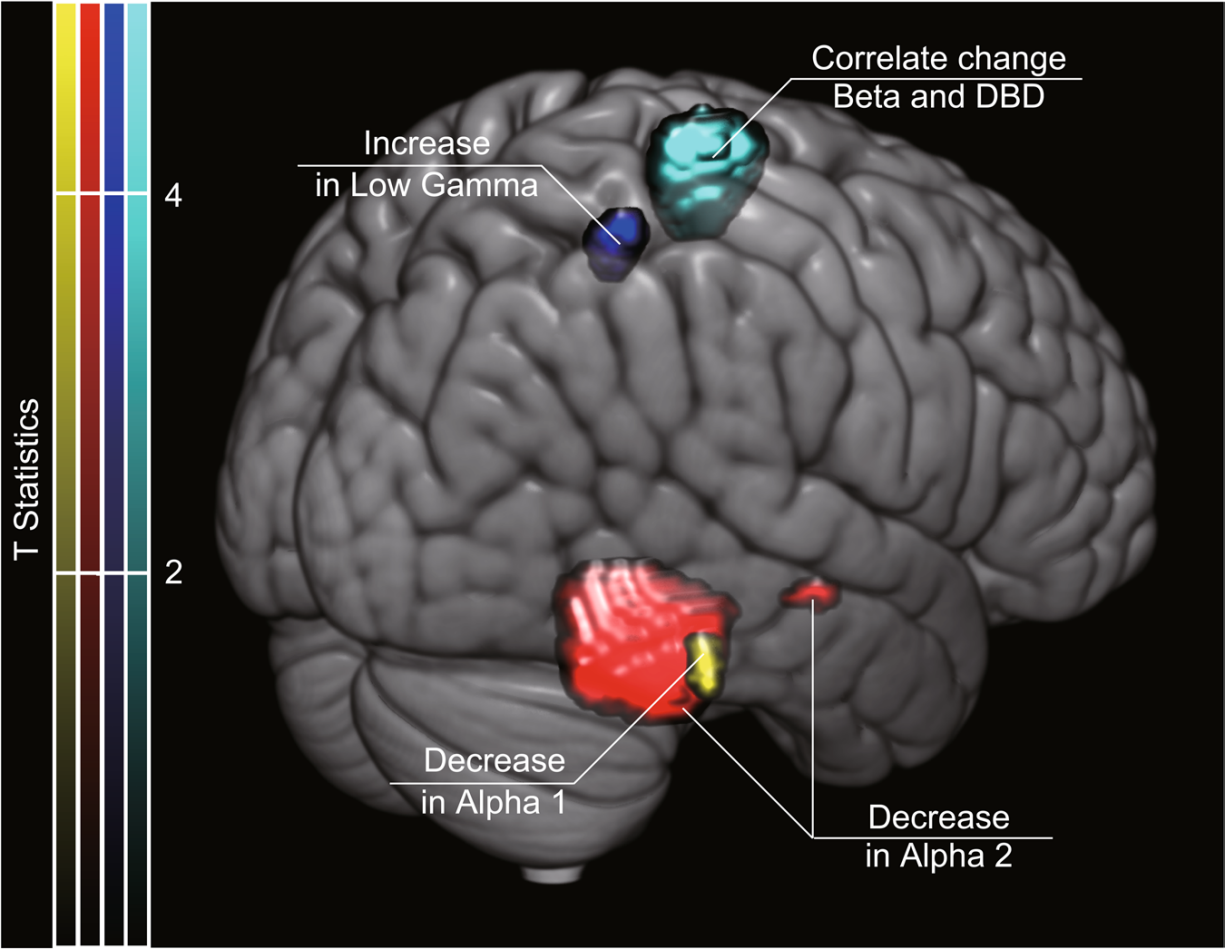
Brain regions with changes in source intensity after the NPT period. Red and yellow areas represent regions in which source intensities decreased after the NPT period. The blue area represents the region with an increase in source intensity after the NPT period. The area in cyan represents the region in which the change in source intensity was positively correlated with the change in the DBD-13 score. The 3D image was created using MRIcroGL (https://www.mccauslandcenter.sc.edu/mricrogl/).

The source intensities were compared between the two scans separately at each frequency band. The alpha1 and alpha2 intensities decreased at the second scan in the right temporal lobe (alpha1: *p* = 0.048, alpha2: *p* = 0.049 at the cluster-level), whereas only the alpha2 intensity exhibited a decline in the right fusiform gyrus (*p* = 0.028 at the cluster-level) (Fig. 1 and Table 2). Moreover, following the NPT period, the low-gamma intensity in the right angular gyrus was increased (*p* = 0.044 at the cluster-level). The results in all other frequency bands did not reveal any significant changes.

**Table 2:** Intensity changes the resting-state brain activity between the two scans (Corresponding to Fig. 1).

Change	Frequency	Cluster level		Peak level		Coordinate			Brain region
		P(FWE)	kE	P(FWE)	T	X	Y	Z	
Decrease after NPT	Alpha1	0.048	25	0.047	4.63	52	−38	−20	Right Inferior Temporal Gyrus
	Alpha2	0.028	858	0.014	5.58	40	−46	−16	Right Fusiform Gyrus
		0.049	14	0.047	4.78	48	−14	−12	Right Superior Temporal Gyrus
Increase after NPT	Low Gamma	0.044	72	0.032	5.13	42	−52	60	Right Angular Gyrus
Positive correlate with DBD-13	Beta	0.028	504	0.016	5.63	30	−28	70	Right Sensorimotor Area

The p-values were corrected for multiple comparisons by the Family-wise-error (FWE) correction. T, t-value; p, p-value; X, X-coordinate; Y, Y-coordinate; Z, Z-coordinate.

Correlations of the MEG source intensities to the interval between the two scans and the changes in behavioural scores (MMSE-J and DBD-13) were also examined. Changes in beta source intensity close to the sensorimotor area of the right hemisphere were positively correlated with changes in DBD-13 scores (*p* = 0.028 at the cluster-level). No other significant correlations were found between source intensities and the time between the scans or the behavioural scores. These results indicate that patients with reduced beta intensity near the right sensorimotor area showed a decline in behavioural disturbances.

**Figure 2:**
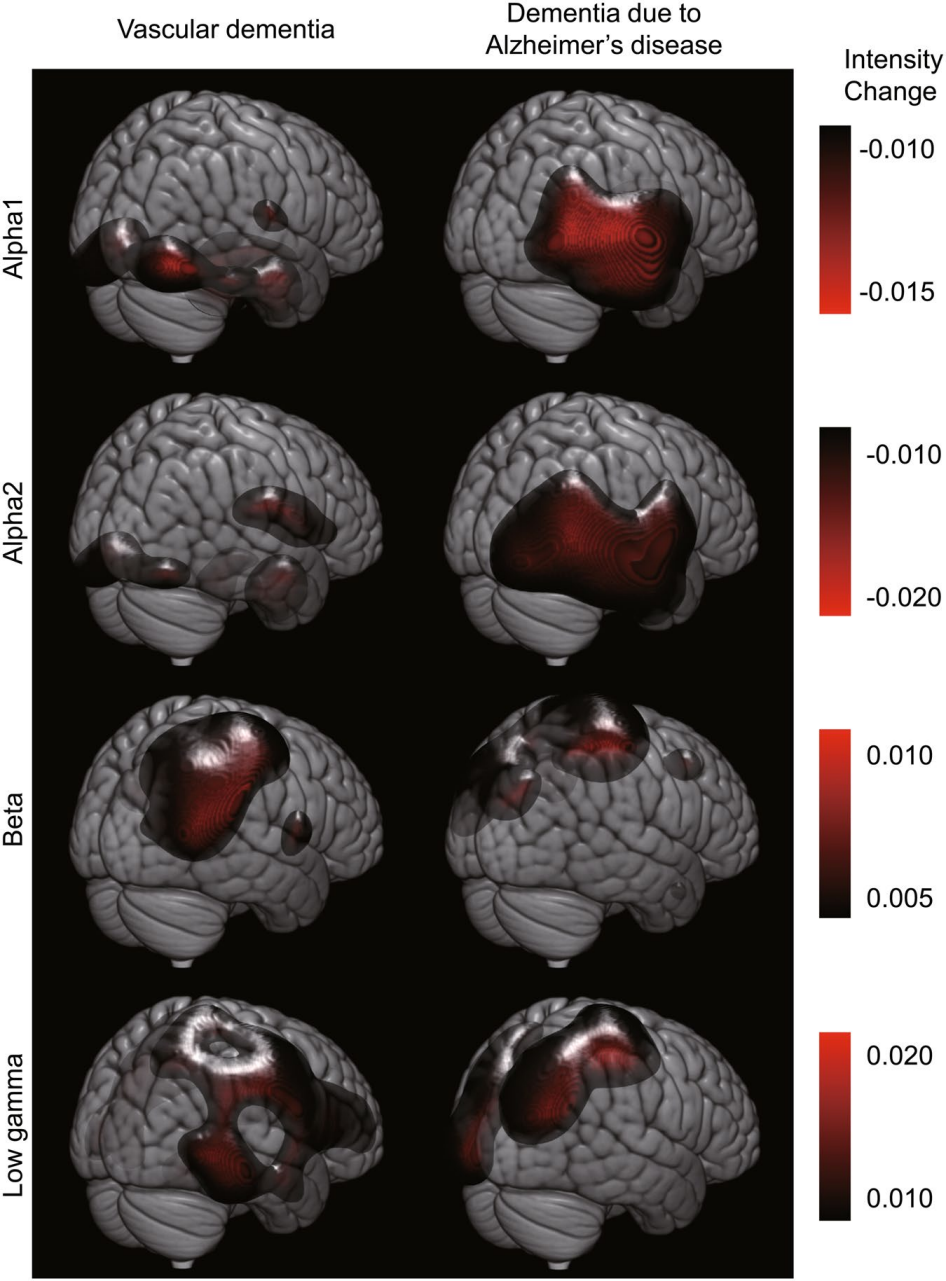
Averaged changes in source intensity for VD and AD (corresponding to Fig. 1). Upper and lower limits of the colour bars correspond to the display threshold. The 3D image was created using MRIcroGL (https://www.mccauslandcenter.sc.edu/mricrogl/).

To determine whether the NPT induced different effects in patients with vascular dementia (VD) and Alzheimer’s disease (AD), we averaged the source intensity changes for patients with VD and AD separately and compared them visually (Fig. 2). Although there were minor differences in changes between VD and AD, they mostly overlapped and shared the same regions as those shown in Fig. 1. Patients with AD showed significant change in source intensity (Table 3); conversely, the changes in the VD were not significant.

**Table 3:** Intensity changes for patients with dementia due to Alzheimer’s diseases (corresponding to Table 2).

Change	Frequency	Cluster level		Peak level		Coordinate			Brain region
		P (FWE)	kE	P (unc)	T	X	Y	Z	
Decrease after NPT	Alpha1	0.201	256	0.001	4.47	56	−38	−24	Right Inferior Temporal Gyrus
	Alpha2	0.083	4942	>0.001	4.28	50	−22	−10	Right Superior Temporal Gyrus
Increase after NPT	Low Gamma	0.242	477	>0.001	3.81	38	−56	60	Right Angular Gyrus
Positive correlate with DBD-13	Beta	0.287	388	>0.001	4.79	26	−24	72	Right Sensorimotor Area

The frequency bands and corresponding brain regions, where the differences reached a statistically significant level, are displayed.

unc, uncorrected; T, t-value; p, *p*-value; kE, cluster size; X, X-coordinate; Y, Y-coordinate; Z, Z-coordinate.

## Discussion

The present study revealed two major findings: (1) NPTs improved cognitive functions in patients with dementia, although behavioural disturbance parameters were not significantly modified, and (2) the spontaneous neural oscillations (i.e. the resting-state brain activity) in the right hemisphere changed over the NPT period and parts of these changes correlated with changes in the behavioural score (DBD-13).

It has been suggested that brains of patients with dementia are severely damaged thus negatively influencing brain plasticity. This is considered to be the reason why these patients do not respond to pharmacological treatments^5^. This idea is supported by the fact that patients with CIND and MCI show improvements in their cognitive functions after NPTs^18–23^, and neuroimaging studies have revealed that these patients still show signs of brain plasticity^16,17^. The present study applied this idea to patients with dementia. We demonstrated that patients with dementia responded to NPT and that the intervention improved the cognitive functions, as reflected by the MMSE-J score. This improvement did not correlate with either age nor initial MMSE-J score, indicating that the potential of NPT is not limited to young patients or patients with mild dementia. A previous study demonstrated that pathological changes are not always in agreement with the clinical symptoms^24^; patients with less brain damage may exhibit more severe dementia symptoms and vice versa. It is, however, reasonable to assume that some patients with dementia have the potential to improve their functions similar to that of patients with CIND or MCI.

Previous studies have shown that NPTs have beneficial effects on healthy older adults^10,25^. The effects of NPTs were independent of the pathological changes in the brain during disease state causing dementia, therefore NPTs are effective in patients with different subtypes of dementia such as VD and dementia due to AD. This is consistent with the novel concept of NPT, which focuses on each patient’s function rather than pathophysiological or neurological causes of their disease^21^. In the present study, we further demonstrated that NPT induced similar changes in the brain activities of patients with either VD or dementia due to AD (see Fig. 2 and Table 3). The primary results (Fig. 1 and Table 2) show the common changes that underlie different pathologies relevant to dementia. NPTs induce neuroplasticity or change the neural network efficiency to compensate for brain functional deficit during disease state^25,26^. A previous study using MRI in healthy older adults showed that NPT changes grey matter densities in the postcentral gyrus, right hippocampus, and superior temporal gyrus^27^. The grey matter density in the postcentral gyrus is modulated by NPT^27^ and the extent of these changes correlate with the NPT outcome. The postcentral gyrus is a part of the sensorimotor area where neural activities are affected by ageing and dementia, and where changes are modulated by and associated with subcortical changes^28^. In the present study, changes in beta intensity in the sensorimotor area correlated with the observed changes in the scores measuring behavioural disturbances. Beta intensity is associated with gamma-aminobutyric acid (GABAergic) neuronal activities^29^ which is correlated to BPSD^30^ and ultimately, neural plasticity^31^. It is plausible that changes in beta intensity in the sensorimotor area reflect the changes in neuroplasticity or neural network efficiency caused by NPTs through the whole brain network system. Another study using functional MRI in healthy older adults revealed that NPT changes brain activities in a widespread network of frontal, parietal, temporal, subcortical, and occipital regions as well as in the thalamus and the caudate^10^. This is supported by additional studies using functional MRI in patients with MCI showing that NPTs modify brain activities within a large network that includes the frontal, temporal, and parietal areas^12^. In the present study, the resting-state activity of the alpha band was altered in the right temporal lobe and the right fusiform gyrus. A larger part of the alpha activity is produced by reciprocal connections between the neocortex and the thalamus^32,33^. Previous studies indicate that NPTs modulate large neural networks including the thalamus^10,12^. The temporal lobe and the fusiform gyrus are anatomically close to the hippocampus and the right hippocampus has been implicated in dementia-related psychosis^34^. NPT effectively reduces BPSD^35^, thus it is a reasonable expectation that NPT alters the spontaneous neural oscillation of the alpha band in the right temporal lobe and the fusiform gyrus. A functional MRI study has shown that NPTs induced brain activity changes in the right angular gyrus and the anterior portion of the left lingual gyrus of patients with AD^36^. Our result showed that gamma intensity in the right angular gyrus increased after NPT. The angular gyrus is one of the brain network cortical hubs^37^ which influences various cognitive functions. It is located in the parietal cortex where ageing and dementia bring about changes at an early stage (Retorogenesis theory)^38,39^. Lesions in the angular gyrus cause symptoms similar to those in BPSD^40,41^. Gamma activity is also correlated with the activities of GABAergic neurons^42^, and a reduction in GABA is associated with poor cognition^43,44^. We speculate that changes in gamma at the angler gyrus reflected the change in neural network efficiency induced by NPTs to compensate for the functional deficit.

In the present study, we used MEG data instead of MRI or functional MRI data. MEG is a non-invasive neuroimaging technique that is sensitive to changes in brain activity related to dementia^45–47^ as it can detect synaptic transmission underlying macroscopically observable brain activity^48^. In dementia research, MEG demonstrated various advantages over MRI. In practice, MRI scans are often difficult and risky for patients with dementia^36^. For example, patients with dementia unintentionally bring metallic items into the scanner at times, which is highly dangerous due to the strong magnetic field produced by an MRI scanner. It takes up to 10 s to abort a scan and rescue the patient in the case of an emergency. Moreover, patients are often not happy with an MRI scan as the device creates loud noises. The patients also feel nervous in the confined space of the MRI gantry and must keep still inside the scanner without having any company. By contrast, a MEG generates neither a magnetic field nor noise. Patients are not opposed to this technique as they are not physically restricted by the scanner and another person can attend to them during the scan to ensure their safety. A scan is completed within 10 min or less including preparations, can be immediately aborted anytime, and in case of an emergency, the patient can be taken out within 10 s. Additionally, MEG scans provide clinicians with an opportunity to detect epileptic discharges. Of patients with dementia, 10–22% have epilepsy as a secondary diagnosis and it is not easy to diagnose epilepsy in older adults because their seizures are often non-convulsive^49–52^. In fact, in the present study, 2 out of the 21 patients presented epileptic discharges during the scan and were excluded from further analyses so that they may be treated with anti-epileptic drugs. In summary, the present study restated that MEG is an ideal neuroimaging technique for dementia studies.

This study has five limitations: (1) The group of patients with dementia was heterogeneous in terms of their pathologies of the brain diseases causing dementia (e.g. stroke or Alzheimer’s diseases), thus making it challenging to fully evaluate how NPTs affected pathological changes of the diseased brain. However, as shown in Fig. 2 and Table 3, NPTs effects are independent of pathological changes and thus highlight the NPT’s common effect on dementia in general, which is a heterogeneous syndrome. (2) We did not address the effects of inter-individual factors such as patient’s age, sex, genotypes, brain diseases causing dementia, and batteries of NPTs. This study aimed to determine whether NPTs changes brain activities (not pathology) and focused on the intra-individual relationship between cognitive/behavioural scores and spontaneous neural oscillations. Contributions of inter-individual factors should be addressed in future studies. (3) We did not include control participants since this was an observational study at a geriatric health services facility. It might be possible that the changes in resting-state activities were caused by spontaneous recovery rather than by NPT. However, the intensity changes in beta-band activity over the sensorimotor area correlated with the changes in DBD-13 scores and the duration of the NPT did not have any significant effects on resting-state brain activities. Therefore, it seems plausible that the changes in the resting-state brain activities were caused by the NPT. (4) The sample size is limited. We avoided generalising our results to NPTs in patients with all kinds of dementia. What we have demonstrated here is that some patients with dementia have the potential to improve their brain functions and resting-state brain activities following NPTs and is largely consistent with previous studies investigating brain plasticity in older adults and patients at risk of dementia. (5) The change in oscillation intensity did not correlate with the change in cognitive scores (MMSE-J). MMSE-J consists of 11 major items targeting different cognitive functions (temporal orientation, spatial orientation, immediate memory, attention/concentration, delayed recall, naming, verbal repetition, verbal comprehension, writing, reading a sentence, and constructional praxis). Each patient changed their scores in different items. In addition, the change in the total score of MMSE-J may not reflect cognitive improvement linearly. The change from 25 to 26 could be bigger than that from 15 to 16 as a change in cognitive function. These two changes could be based on neurological changes in different cortical areas. We can assume that the heterogonous improvement and non-linearity prevented us from observing a statistically significant correlation between changes in the oscillation intensity and the MMSE-J sore.

Taken together, NPTs can change the resting-state brain activity and improve dementia symptoms. These changes are based on neural plasticity and not limited to patients in pre-dementia conditions (CIND and MCI). Clinicians and therapists should pay more attention to primary care patients with dementia because these patients have the potential for improvement with NPTs.

## Methods

### Patients, rehabilitation procedure, and ethics

A total of 21 patients with dementia (12 females, 9 males; age, average ± standard deviation [SD]: 85.4 ± 5.4 years) were enrolled from our geriatric health services facility ‘Kakehashi’ regardless of the brain disease causing dementia (e.g. stroke or AD). The group of patients with dementia was an epitome of the population of dementia in the region. The dementia diagnosis was established by clinicians prior to patients’ admission to the facility. Most patients were affected by AD with other types of dementia also being present in the study population (see Table 1). Medication schedules established prior to admission of the patient to our facility were continued unchanged throughout the NPT period in the facility, if possible. Usually, patients stayed in the facility for several months (average ± SD: 83.9 ± 32.7 days, ranging from 30 to 147 days) where they were treated every day by the NPT therapists. NPT consisted of five major activities: (1) physical exercise (e.g. regular daily activity training or ergometer cycling), (2) therapeutic role-playing (e.g. reminiscence therapy), (3) nursing care (e.g. proper eating, drinking and keeping sanitary environment), (4) horticultural therapy, and (5) self-cognitive training (e.g. colouring books or crossword puzzles). All patients participated in the activities (1) – (3), whereas (4) and (5) were optional activities. The battery of these NPTs and the details of the activities were individually adjusted daily by the NPT therapists after considering patients’ physical, mental, and cognitive conditions. The length of the daily NPT programmes [i.e. (1), (2) and (4)] was in accordance with the Japanese regulations: 20–40 min, depending on the patients’ condition. During the patients’ stays in the facility, dietary and hygienic regimens were provided by nurses, dental hygienists, and nutritionists. All patients with their families gave written informed consent to participate in this study. This study was conducted in accordance with the Declaration of Helsinki and was approved by the Ethics Committee of Hokuto Hospital.

### Behavioural assessments

**Figure 3:**
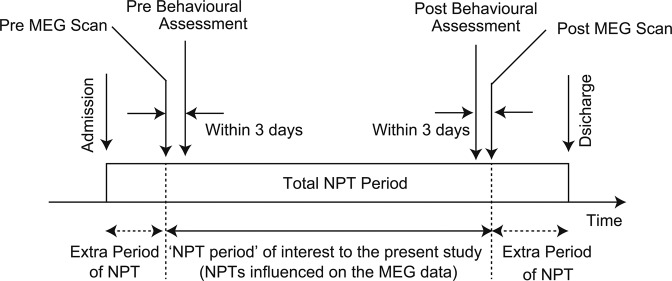
Time course of the present study. MEG scans and behavioural assessments and were carried out two times during non-pharmacological treatment (NPT) period. Each MEG scan and its corresponding behavioural assessments were obtained within 3 days. The interval between the two MEG scans was defined as the NPT period. Some patients were involved in ‘extra’ NPT programmes (before their first MEG scan and/or after the second MEG scan), it is not relevant to the present study.

The levels of cognitive impairments and behavioural disturbances in patients were assessed by therapists using the Japanese version of the Mini-Mental State Examination (MMSE-J)^53,54^ and a short version of the Dementia Behaviour Disturbance Scale (DBD-13)^55^. Each patient completed a set of behavioural assessments (MMSE-J and DBD-13, conducted on the same day) two times during NPT period (Fig. 3). The interval between the two tests was several months (average ± SD: 74.7 ± 32.1 days, ranging from 26 to 132 days). The MMSE-J is a 30-point cognitive test. In this test, a lower score indicates a more severe cognitive impairment^56^, and dementia is suspected when the score is below 23. The DBD-13 is a 52-point behavioural rating scale and consists of 13 items (item #1, 2, 3, 4, 5, 15, 16, 17, 21, 24, 26, 27, and 28 of the original version of the Dementia Behaviour Disturbance Scale^57^) and a lower score indicates a less problematic behaviour (such as agitation, aggression, anxiety, or apathy). There is no reference value for the DBD-13.

### MEG scanning

All patients visited our MEG centre at Hokuto Hospital two times during the NPT period (Fig. 3). The 3-minute travel was comfortable by car between the geriatric health services facility and the MEG centre. The interval between the two scans was a few months (average ± SD: 75.0 ± 29.7 days, ranging from 28 to 133 days). Each MEG scan and its corresponding behavioural assessments were obtained within 3 days. The two intervals between the two behaviour assessments and two MEG scans were almost identical. The interval between the two MEG scans was defined as the NPT period. In the present study, some patients were involved in the NPT programmes before the first MEG scan, while others were involved after the second MEG scan; however, these “extra” periods were not considered in our study (Fig. 3). Spontaneous neural oscillations (i.e. resting-state brain activity) were recorded for 5 min using a 160-channel whole-head type magnetoencephalography system (MEG vision PQ1160C; Yokogawa, Kanazawa, Japan). During the scan, patients were asked to calmly stay in the supine position with eyes closed in a magnetically shielded room. The scanning condition was controlled to be as consistent and comfortable as possible. For safety reasons, another person remained next to the patient. The sensor and reference coils were gradiometers of 15.5 mm in diameter and 50 mm in the baseline, and each pair of sensor coils was separated by a distance of 23 mm. The sampling frequency was 1,000 Hz with 200 Hz low-pass filtering during the recording. To co-register MEG source images with structural brain images acquired by standard magnetic resonance imaging (MRI), three fiducial magnetic marker coils were placed on the patients’ face (5 mm above the nasion and bilaterally 10 mm in front of the tragus) during the MEG scan.

### MEG analysis

Of the 21 patients, 3 patients were excluded from analysis: 2 exhibited epileptic discharges during the MEG scans and 1 induced severe artefacts due to dyskinesia. The remaining 18 patients were analysed offline using the software package SPM-12 (Wellcome Trust Centre for Neuroimaging, London, UK; https://www.fil.ion.ucl.ac.uk/spm/) and the MEAW system (https://www.hokuto7.or.jp/hospital/lang/english-home/meaw/) (Table 1). For the ease of analysis, the continuous MEG signals were divided into 10 s segments. Epochs in which the magnetic signal exceeded 6,000 fT, were discarded. Since the experimental environment generated a utility frequency, a 50-Hz band-stop filter was applied to the epoched data. These filtered data were directly used for source-level analyses. To identify the locations of the brain producing the resting-state-induced component, the source inversion procedures were applied to the oscillation components of delta (0–3 Hz), theta (4–7 Hz), alpha1 (7–9 Hz), alpha2 (9–11 Hz), alpha3 (11–13 Hz), beta (13–25 Hz), and gamma (low gamma, 26–40 Hz; high gamma, 41–80 Hz) separately, using a maximal smoothness algorithm with a spatially coherent sources model (i.e. COH algorithms implemented in SPM-12)^58^, which is comparable to sLORETA^59^. The COH algorithm is a popular source inversion algorithm and is often used in clinical environments^60,61^. Forward modelling was performed for the whole brain using a single shell model with canonical MR images provided by SPM-12. The source inversion and estimation were performed by applying filters which corresponded to each frequency band (from delta to high gamma). No source priors were used for source estimation. The source images were smoothed (20 × 20 × 20 mm) and taken to the second (group)-level analysis.

Two types of the second (group)-level analyses were carried out: (1) to find brain regions in which intensities of the spontaneous neural oscillation were different between the two scans, and (2) to find brain regions in which changes in source intensities were correlated with changes in MMSE-J scores, DBD-13 scores, or the time between the two scans. For the first analysis, the source images were compared within each patient using the paired *t*-test at each frequency band with the three covariates MMSE-J scores, DBD-13 scores, and the time between the two scans. Both positive and negative effects of these covariates on the source intensities were evaluated by building *t*-contrasts with +1 and −1. For the second analysis, source images were created to represent changes in source intensities between the two scans using Image Calculator, which is an SPM-12 tool. These images were analysed using the one-sample *t*-test with a covariate of the change in the scores of MMSE-J or DBD-13 or the time between the two scans. Here, we report the source locations of peak level activations at a significance threshold of *p* (corrected for family-wise error [*FWE*]) = 0.05 and a cluster extent at k > 10 (=80 mm^3^)^62^. Cortical areas at which the peaks of the estimated sources were located were identified using the SPM-12 software.

After the main analyses, we performed two additional analyses to determine whether the NPT induced different effects on patients with VD (n = 4) and AD (n = 11). For the first step, the changes in source intensities between the two scans were averaged over patients with the same pathological labelling (VD and AD, separately) using the Image Calculator; the averaged images were visually inspected (Fig. 2). For the second step, for both the VD and AD patient groups, the source intensities were compared within each patient using the paired t-test for each frequency band (the procedures are same as the main analysis described above). Here, we report the source locations of peak level activations at a significance threshold of *p* (uncorrected) = 0.001 and a cluster extent at k > 10.

### Statistical analysis

Bootstrapping approaches were used to evaluate changes in MMSE-J and DBD-13 scores over NPT period considering a relatively small number of patients (n = 18). First, changes in these scores between pre- and post-NPT were evaluated. The difference between the pre- and post-NPT scores was resampled with 10,000 times replacement across the 18 patients, and the percentage of the resampled differences, being larger or smaller than 0 (the smaller value), was taken as the significance level. Second, the correlations between the patients’ age, the duration of the NPT period, and the MMSE-J and DBD-13 scores were evaluated. For each pair of variables, the correlation coefficient was calculated from resampled with replacement data across the 18 patients for 10,000 times, and percentage of the resampled coefficients, being larger or smaller than 0 (the smaller value), was taken as the significance level. Statistical analyses were performed using MATLAB (MathWorks, MA, USA).

## Data Availability

The datasets generated during and/or during the current study are available from the corresponding author on reasonable request.
